# Page for the General Public

**DOI:** 10.12945/j.aorta.2017.17.089

**Published:** 2018-09-24

**Authors:** Anneke Damberg, on behalf of the Editorial Office

The following pages summarize and review this issue’s articles for an audience without a background in medicine or research.

## Original Research Articles

*Paul C. Tang et al.: “Coronary Events in Patients Presenting for Repair of Acute Type A Aortic Dissection“*

When a patient undergoes emergent surgery for type A aortic dissection, in which a tear occurs in the layers of the body’s main artery, there is usually no time to perform an additional study of the state of the patients coronary arteries, the vessels which supply the heart muscle with blood. The authors of this study analysed how often these patients also have disease of their coronary arteries and how often this causes problems. Studying 31 patients with aortic dissection and 123 patients who underwent planned surgery for aortic aneurysm (dilation), they came to the conclusion that coronary heart disease was missed only in very few patients, and that not screening for it before surgery did not decrease survival due to non-aortic causes. They therefore conclude that it is not necessary to delay emergent surgery for aortic dissection to perform coronary angiography to screen for coronary heart disease. However, due to the design of the study and limited patient numbers, further and larger investigations are necessary.

*Andrew G. Sherrah et al.: “Multi-Velocity Encoding Four-Dimensional Flow Magnetic Resonance Imaging in the Assessment of Chronic Aortic Dissection“*

Patients who suffered aortic dissection, in which a tear occurs in the layers of the body’s main artery, need to undergo follow-up imaging after surgery to see how the disease progresses. The authors investigated a new imaging method using MRI (magnetic resonance imaging), in which they do not only use steady images but also visualize blood flow dynamics. Blood flow patterns and pulsations were studies in 10 patients with dissection and 9 healthy subjects. There were significant differences in blood flow patterns between healthy patients and patients with aortic dissection. This imaging technique could be helpful in the follow-up of patients with aortic dissection, even though its significance and usefulness needs to be further studied.

## Case Reports

*T. Joseph Watson et al: “Acute Bilateral Lower Extremity Paralysis Secondary to Acute Thrombosis of an Infrarenal Abdominal Aortic Aneurysm“*

*Watson et al.* Describe a case of a patient with an abdominal aortic aneurysm, a dilation of the body’s main artery in the abdomen, which was suddenly occluded by blood clots which led to paralysis of the legs. The patient underwent open surgery to replace the aneurysm and reopen the clotted vessels. Overall, it is common that blood clots develop in an abdominal aneurysm but they rarely lead to occlusion of blood flow. The paralysis is mainly due to impaired blood flow to the spinal cord. Patients need to receive medication to impair blood coagulation and undergo urgent surgery to restore blood flow.

*Michal Nozdrzykowski et al: “Thoracic Endovascular Aortic Repair for Aortoesophageal Fistula After Covered Rupture of Aortic Homograft: A Durable Option?“*

The authors report a case of a patient who had to undergo emergent surgery to replace parts of her aorta, the body’s main artery. Three years later, she developed an aortoesophageal fistula. In this rare but serious complication, a connection between the aorta and the esophagus develops, which can cause life threatening bleeding. Most likely, the complication was due to an infection. The patient was operated on again and her aorta replaced with a piece of donor aorta. When the complication recurred, it was treated with a stent graft, a prosthesis inserted into the vessel via a vessel in the groin, and repair of the esophagus. The postoperative course was complicated, and when an infection recurred one year later surgery was deemed too dangerous and the patient discharged on palliative care. The authors discuss several treatment options for an esophageal fistula. With regard to using stent grafts, they only see it as a bridging option to definitive surgery or in patients with poor general condition, mainly due to the high risk of infection.

## What I Did

*Kaufeld et al.: “Left Ventricular Intussuception of an Intimal Flap in an Aortic Dissection Type A.“*

*Kaufeld et al* . describe the case of a patient who had a type A aortic dissection, in which a tear occurs in the layers of the body’s main artery very close to its origin at the heart. In this case, the tear involved the whole circumference of the aorta. In this rare case, the flap of the inner layer even protruded into the heart itself, causing severe leakage of a heart valve, the aortic valve. The patient underwent surgical repair and recovered quite well, even though a preoperative stroke caused lasting speech impairment.

